# Violations of workers’ rights and exposure to work-related abuse of live-in migrant and live-out local home care workers – a preliminary study: implications for health policy and practice

**DOI:** 10.1186/s13584-018-0224-1

**Published:** 2018-06-21

**Authors:** Ohad Green, Liat Ayalon

**Affiliations:** Bar-Ilan University, Ramat Gan, Israel

**Keywords:** Home care, Migrant workers, Abuse, Workers’ rights, Older adults, Immigration, Long-term care, Living conditions

## Abstract

**Background:**

Home care workers work in an isolated environment, with limited supervision and guidance which makes them more prone to abuse and exploitation. While past research focused mostly on the well-being of care recipients, this study aimed to shed light on the care workers’ daily reality and explore if and how boundaries of professional care work are blurred. Our primary aim was to assess the working conditions and the prevalence of abuse and exploitation among live-in migrant home care workers and live-out local home care workers.

**Methods:**

A random stratified sample of Israeli older adults aged over 70, who are entitled by law to home care services was used to recruit 338 migrant live-in home care workers and 185 local live-out home care workers to a face-to-face survey. The participants were asked about their relationship with the care recipient and their exposure to violations of workers’ rights and work-related abuse.

**Results:**

Almost all the participants reported exposure to certain workers’ rights violations. Among the migrant live-in care workers, it was found that 58% of them did not receive any vacation days besides the weekly day-off, about 30% reported not get even a weekly day-off on a regular basis, and 79% did not get paid sick days. Local live-out care workers also suffered from a high prevalence of exploitation - 58% did not get any vacation days besides the weekly day-off, and 66% did not get paid sick leave. 20% of the local live-out care workers, and 15% of the migrant live-in care workers did not receive a signed contract. A smaller portion (7.4% among migrant care workers, 2.5% among local care workers) reported work-related abuse. When compared to local workers, migrant home care workers were more vulnerable to some worker’s rights violations, as well as emotional abuse.

**Conclusion:**

These findings are disturbing, as work-related abuse and exploitation affect not only the well-being of the care worker but also the health of the care recipients, as the quality of care provided deteriorates. At the public policy level, more significant attention and regulation of the home care industry is needed. The frequency and the nature of home visits made by home care agencies must be changed. Also, home care workers should be offered emotional support.

## Background

The world population is ageing, and the number of older adults who need assistance in activities of daily living has been increasing accordingly [1]. At the same time, the ability to provide informal care to frail older adults within the family is declining, among other things due to drop in fertility [2], women joining the workforce [3] and increasing divorce rates [4]. Consequently, in most western countries the home care services are provided by paid care workers - either locals or migrants [5]. This solution is a win-win situation for both care recipients and governments. For the older adult, it allows them to stay in their homes as long as possible, as most of them hope and aspire [6]. For governments, every day at home means a day less in public funded expensive long-term placement [5].

While this arrangement is financially cost-effective, it entails other costs. Home care workers are particularly vulnerable to both violations of workers’ rights and work-related abuse. Violations of workers’ rights refer to disregarding rights that relate specifically to being a worker [7], whereas work-related abuse refers to any violent acts against a person at work or on duty [8]. The vulnerability of home care workers stems mainly from the intimate nature of their work, and the long-term and intensive relationships between the care recipient and the care provider.

Migrant home care workers might be at greater risk for exploitation and work-related abuse compared with local home care workers, as they are in the middle of the “three axes of disadvantage” [9]. In addition to the intimate nature of their job and the gendered aspect of care work, their vulnerability stems from their migratory status as temporary visitors [9]. As a result, their duties and rights are different from that of citizens [9]. Moreover, many migrant care workers pay thousands of dollars to obtain a work permit in the host country [10, 11]. Thus, in their first years of employment, most of their salaries are used towards settling these enormous debts. Under these circumstances, leaving an abusive employer is extremely difficult [10].

### The Israeli case

Whereas in many western countries such as the US and the UK, the support offered by the state to older adults is limited, and is mainly given in extreme cases of need [12], the Israeli government offers relatively generous support, in order to assist family members who care for older adults in their homes. In the home care sector, there is no governmental limit to the number of working permits provided to migrant workers, as it is determined solely on the basis of demand. Thus, while in Western countries migrants in the care sector constitute between 18 to 25% of the care work for older adults [13], in Israel about 50% of the care work for older adults is provided by migrant workers.

The government supports Israeli citizens aged 60 (females) or 65 (males) and up, who live in their homes and are unable to perform their activities of daily living (such as eating or bathing) independently. The result of this arrangement is that most older adults with functional impairments live at home, and only a small percentage lives in long-term care facilities [14]. This arrangement is cost-effective, as the cost of paid home services is far lower than the price of long-term care facilities [15]. This is also the preferred option of the older adults and their family members, who wish for the older adult to stay at home for as long as possible [6].

The Israeli home care system offers two options for in-home care services for older adults with functional impairments who wish to stay in their homes: Live-out home care services and live-in home care services. Live-out home care service is given to older adults with mild to moderate impairments in activities of daily living (ADL). Older adults might be entitled to partial support (up to 9.75 h per week) or “full” support (22 h per week) [16], depending on degree of impairment. Live-out care service is provided only by locals, i.e., Israeli citizens. There are about 70,000 local home care workers and most of them work part time only (average of 23 h per week) with multiple older adults [6]. The local live-out care workers tend to view their job as a low-status one, and continue doing it because no other job opportunities are available to them [17].

Live-in home care services are provided only by migrant workers. Live-in home care services are provided to older adults who need round-the-clock care. These individuals are severely impaired in their activities of daily living, or need constant supervision due to cognitive impairment. Currently, there are about 48,000 migrant workers who work as live-in care workers legally, and approximately 12,000 who work illegally [18]. The majority of the migrant care workers are Filipino women [19] as in other developed countries such as the UK, US and Canada [20]. Israel constitutes an important case study, as the country with the second largest ratio of migrant care workers to citizens within the OECD countries, after Italy [16].

As in other countries [21], the social rights and workers’ rights of migrant care workers in Israel are restricted. For example, they are entitled to medical insurance, but at the same time, are excluded from other Israeli employment laws which would have ensured their right to overtime payment. This is similar to the US, the UK and Canada, where some of the regulations for institutional settings do not apply to migrant home care workers [20]. Nonetheless, and similar to the US [22], there are a few rights that are not linked to citizenship or residency, such as the right to a minimum wage and the provision of sick leave which apply also to migrant home care workers. Whereas in countries such as the US or the UK, the support offered by the state to older adults is limited [12], and sometimes imposes bureaucratic hurdles [20], in Israel, this process is smoother [23]. Every older adult, who is severely impaired in activities of daily living, or needs constant supervision, is allowed to hire a round-the-clock migrant home care worker through a generous subsidy by the state (about 70%).

Although the process might be less bureaucratic for the older care recipient, this is not the case for the migrant care worker. As in many other countries, migrant home care workers need to go through a long process to get a work permit [24] and need to pay thousands of dollars in illegal fees for brokers in the host and sending countries. Although the law allows the home-care companies to collect a maximum of 1100 USD [24], in 2016, the average amount paid by migrant care workers in Israel was about 10,500 USD [25]. These amounts are considered enormous in their country of origin. As a result, the migrant workers often are forced to borrow money from their families or communities. During the loan repayment period, many migrant care workers will do anything in their power to maintain their place of work, sometimes at the cost of tolerating serious exploitation and even sexual or physical violence [25].

Social workers can play an important part in securing the welfare of care workers [26]. Many services related to home care are coordinated, provided and supervised by social workers. Monitoring the psychosocial needs of older adults and helping to adjust to chronic conditions [27] are among the roles of social workers in the home care setting. In addition, some social workers are responsible for the initial placement of the home care workers within the home and for the welfare of the care recipient as well as the care worker. As such, gaining knowledge into the working conditions of home care workers is crucial for social workers. Because this is an underserved population, social workers might serve this population and support its basic needs for welfare and emotional support.

### The present study

As the developed world becomes increasingly dependent upon formal home care, we must make sure that the rights of those who carry out this demanding job are honored. Without these basic rights, the physical and mental states of the care workers are likely to deteriorate and so is the quality of care provided [28]. We aimed to explore the working conditions of both local live-out and migrant live-in care. To our knowledge, this is the first study to do so. Most of the research on work-related abuse of health care workers has focused on institutions and hospitals [29–31], and less attention has been given to the home care environment [32, 33]. Our second aim was to see whether there are differences between local live-out and migrant live-in care workers in terms of violations of workers’ rights and exposure to work-related abuse. Moreover, this is the first study to be based on a representative sample. It is important to note that the study is focused only on migrant home care workers who have a legal permit because this is the only way to obtain a representative sample in Israel. This population is likely very different from migrant workers without a permit and is expected to be more similar to local workers in terms of its employment rights.

## Methods

### Procedure

**Fig. 1:**
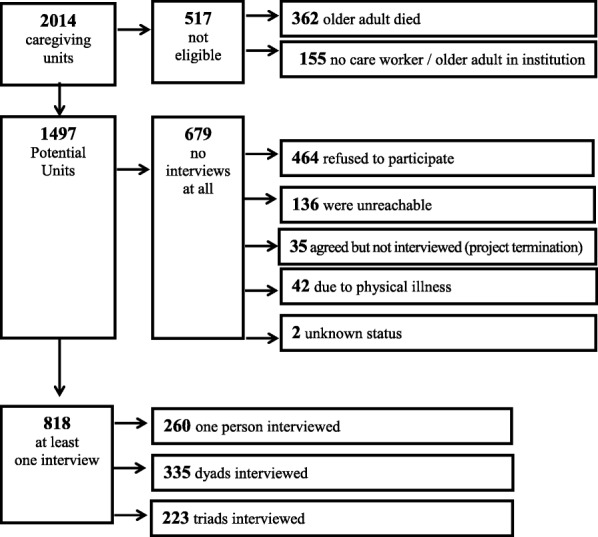
Sample flow

**Fig. 2:**
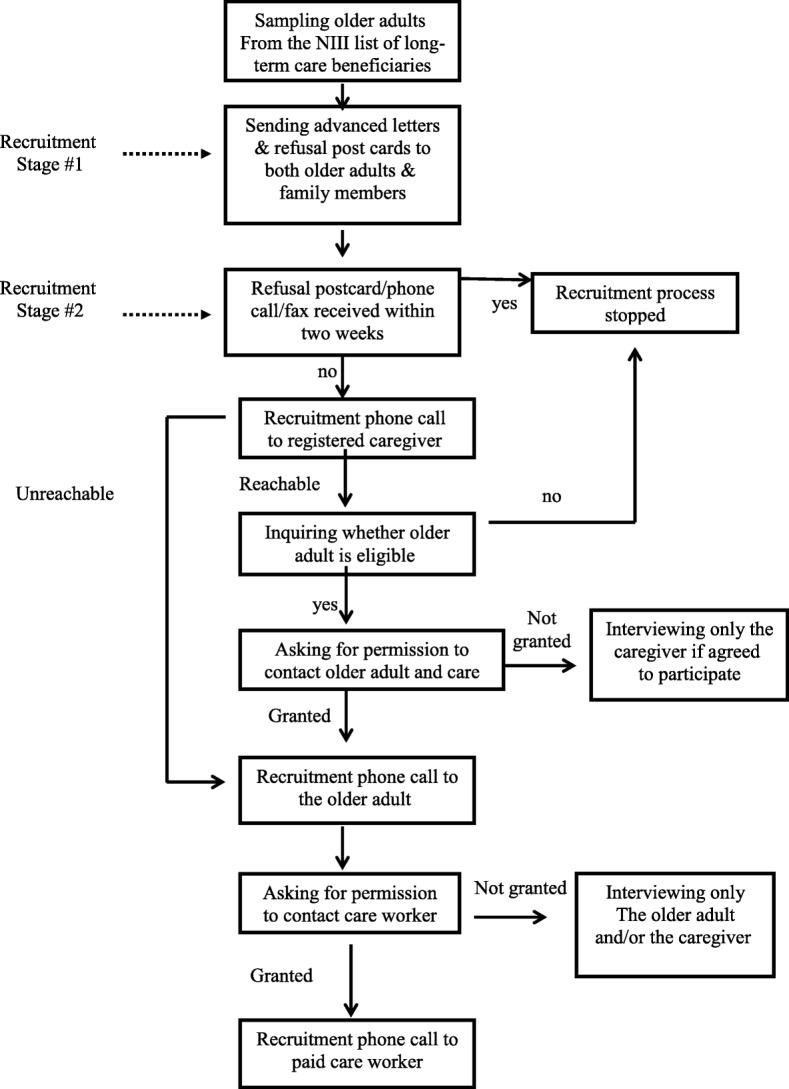
Recruitment process

This study was part of a research project regarding home care services for older adults. It was funded by the National Insurance Institute of Israel (NIII) and approved by the ethics committee of the authors’ University. As such, the recruitment focused on the “caregiving unit”, which was composed of an older adult, his or her family member and the care worker (either local or migrant). A random stratified sample of older adults over the age of 70 who lived in central Israel was drawn from a national pool of older adults who received financial assistance from the NIII under the long-term care community law. The home care workers were recruited indirectly, with the help of the older care recipient or his or her family members. A preliminary letter was first sent to a group of randomly chosen 2014 older adults and their primary family caregivers. Two weeks later, a recruitment phone call was placed to those who did not refuse further contact by phone, fax, or a pre-paid envelope sent to them. If the family member and/or the older adult confirmed that they were willing to participate, we asked them for the phone number of their home care worker, in order to invite this person to participate in the study. We chose the face-to-face method as some of the older adults are incapable of filling out a self-administrated questionnaire. For comparability reasons, this method was used also for the care workers. During the recruitment process, it was emphasized that all potential participants had the right to refuse participation in the study and may withdraw from the study at any time. The participants were explicitly told that their answers would be anonymous and confidential and that refusal to participate would not harm them in any way. Israeli home care workers were interviewed only if they were Hebrew-speaking. Migrant home care workers were interviewed only if they were English or Russian-speaking. The sample flow and the recruitment process are presented in Figs. 1 and 2, respectively. For a full description of the procedure and recruitment methods please refer to Ayalon L, et al [6].

### Participants

In all, 338 migrant live-in care workers and 185 local live-out care workers completed the questionnaire (see Table 1).

**Table 1:** Demographic comparison

	Migrant care workers *N* = 338	Local Care workers *N* = 185	t/χ^2^
Gender (% Females)	84.0%	92.2%	χ^2^ = 3.84, *n.s*
Age	38.86	53.12	χ^2^ = 9.54, sig < .01
Marital status (% Married)	57.7%	56.2%	χ^2^ = 0.11, *n.s*
Years of Education	11.41	12	*t* = −1.88, *n.s*
Financial status			χ^2^ = 66.16, sig < .001
Can’t make ends meet	13.2%	42.1%	
Have just enough to get along	50.8%	44.8%	
Comfortable	28.8%	12.0%	
Excellent	7.2%	1.1%	
Level of Hebrew (0–5)	2.24	3.69	*t* = −15.47, sig < .001
Years with current care recipient	2.73	2.53	*t* = .89, *n.s*

Migrant workers - Most migrant home care workers were women (84%), and the mean age was 38.86 (SD = 8.56). About 58% of them were married, and the majority (62%) were high-school graduates. 13% stated they do not make ends meet, while 7% rated their financial status as excellent. Their average level of Hebrew was 2.24 (see Table 1).

Local live-out care workers - 92.2% were women, 56.2% were married, and about 70% were high-school graduates. The mean age was 53.12 (SD = 10.61). 42% stated they do not make ends meet, while only 1% rated their financial status as excellent. Their average level of Hebrew was 3.69. 60% of the local live-out care workers were not born in Israel. The majority of them were born in the former Soviet Union (40%), and the rest (20%) were born elsewhere.

### Measures

#### Socio-demographic characteristics

The participants were asked about their age, gender, duration of stay in Israel, years working as a home care worker and subjective financial situation (range: “can’t make ends meet”(1) to “excellent” (5)).

#### Violation of workers’ rights

The participants were asked whether or not they were given six basic workers’ rights to which they were entitled in accordance with the Israeli law [34] in the past year (e.g., paid sick days, a written contract etc). The question about a weekly day off was relevant only for live-in migrant care workers, as live-out local care workers do not work around-the-clock.

#### Exposure to work-related abuse

The measure was based on Gettman & Gelfand [35] and further adapted based on qualitative interviews with migrant home workers and family members of older care recipients [28]. Sixteen statements that evaluated exposure to different kinds of abuse (sexual, emotional, and physical) were included. Sexual abuse included statements such as “been kissed or touched in a way that made you feel uncomfortable” and “offered money for sex”. Emotional abuse included statements such as “been yelled, shouted, or sworn at”. Physical abuse included statements of direct violence, such as “someone threatened to hit you with a heavy object”. For each of the sixteen possible incidents, the participants were asked to indicate whether the incident had happened or not. If the abuse happened, respondents were asked to indicate who was responsible for the abuse: the care recipient or his or her family members. The three types of abuse were dichotomized to indicate whether abuse in each of these four domains occurred or not. As the level of all types of abuse was very low, we combined the abuse assigned to the care recipient and to the family member.

#### Living conditions

Migrant live-in home care workers were asked whether they had a separate room, a separate bed, a separate closet and free access to their passport. These questions were not relevant in the case of local live-out care workers.

## Results

Table 2 shows the frequency of violations of workers’ rights, work-related abuse and living conditions of live-in migrant and live-out local home care workers. Both live-in migrant and live-out local home care workers were subject to violations of workers’ rights.

**Table 2:** Frequency of work-related abuse and workers’ rights violation among migrant and local care workers

	ALL *N* = 523	Migrant care workers N = 338	Local Care workers N = 185	χ^2^
Violations of workers’ rights				
Did not receive vacation days	57.5%	58.4%	48%	χ^2^ = 4.93, sig < .05
Did not receive paid sick days	76.1%	78.9%	66.3%	χ^2^ = 9.54, sig < .01
Did not receive a written contract	16.2%	15.8%	19.8%	χ^2^ = 1.18, *n.s*
Did not receive the financial compensation they are entitled to	3.1%	39%	2.3%	χ^2^ = 0.95, *n.s*
Required to do more than job requirements	3.3%	27%	5.1%	χ^2^ = 1.9, *n.s*
Didn’t receive a weekly day off		35%	Irrelevant^a^	
At least one violation		89.7%	89.3%	
At least three violations		64.6%	39.5%	
Work-related abuse				
Emotional abuse	9.5%	12.7%	5.4%	χ^2^ = 7.01, sig < .01
Physical abuse	< 1%	1.2%	0.5%	χ^2^ = 0.52, *n.s*
Sexual abuse	< 1%	1.2%	0.5%	χ^2^ = 0.92, *n.s*
At least one abuse		7.4%	2.7%	
Living conditions				
Do not have a separate room		12.3%	irrelevant^a^	
Do not have a separate bed		3.7%	irrelevant^a^	
Do not have a separate closet		5.5%	irrelevant^a^	
No access to passport		2.1%	irrelevant^a^	

^a^Local care workers employed only part-time, only during weekdays, and not living with the care recipient

Among the migrant live-in care workers, it was found that 58% of them did not receive any vacation days besides the weekly day-off. About 30% reported not get even a weekly day-off on a regular basis. 79% did not get paid sick days. About 15% did not receive a contract which state their working conditions, workers’ rights and financial compensation. Work related abuse was less frequent. 12.7% reported emotional abuse, and about 1% suffered from physical or sexual abuse. As for living conditions, most of the migrant home care workers reported having adequate living conditions. However, 12.3% reported not having their own room and 3.7% reported not having their own bed.

The local live-out care workers also suffered from high prevalence of exploitation. 58% did not get any vacation days besides the weekly day-off, and 66% did not get paid sick leave. A very small portion (2.5) reported work-related abuse. About 20% did not receive a contract which state their working conditions, workers’ rights and financial compensationWhen compared to local workers, migrant home care workers were, migrant home care workers were more vulnerable to violations of the provision of paid sick days and vacation days. Live-in migrant home care workers were twice more likely to report emotional abuse compared with live-out local home care workers.

## Discussion

Home care workers are vulnerable to work-related abuse and exploitation [33]. The present study evaluated violations of workers’ rights and exposure to work-related abuse among live-in migrant home care workers and live-out local home care workers. Our study demonstrates that both migrant and local home care workers suffer from violations of workers’ rights. The fact that almost all participants reported at least one kind of violation of workers’ rights during the past year sketches a disturbing picture and underscores the extent to which home care workers are susceptible to exploitation. The unique vulnerability of care workers lies at the intersection between gender, race and social status and dependency [36]. Care work, similar to other forms of “non-reproductive labor” like cleaning and child-rearing, has a racialized history, which makes cares particularly vulnerable [37]. A theoretical framework that has been widely applied is the worldwide ‘care chains’ [38], in which people are connected via the work of care. People who are employed in these jobs are almost always women from the bottom of the social hierarchy, while the recipients will be in a higher class [39]. As Glenn [39] noted, these gaps are used by the employers to justify exploitation. In Israel, both local and migrant workers can be considered as the ‘others’, as most of them are immigrants, former immigrants or are a part of an ethnic minority (Arab-Israeli). In King’s [40] words, as “racism multiplied by sexism multiplied by classism” (p. 47), their demographic profile will probably cause them to suffer from multiple jeopardies. The second layer of vulnerability is composed of situational factors. In contrast to care workers in institutions who enjoys the support of colleagues and social networks, home care workers work in isolation, with little supervision and guidance. Hence, their social networks are limited. Furthermore, because of the intimate nature of the job, the boundaries of professional care work are blurred [41]. Also, the characteristics of live-in home care bring up issues concerning essential workers’ rights. In contrast to live-out home care work, in the case of live-in home care settings, breaks, working hours, and other work conditions are often undefined [42].

The picture that emerges with regard to work-related abuse is different. In contrast to previous research which has shown that home care workers are particularly vulnerable to work-related abuse [32] we found low rates of work-related abuse both among live-out local home care workers and live-in migrant home care workers. Only 9% of the participants reported emotional abuse, the most commonly encountered type of work-related abuse. It is important to note that in contrast to past research, this is the first study to rely on a representative sample. Nevertheless, the fact that the care workers who participated in the study were recruited indirectly, through the care recipients or their families could also account for the findings. It stands to reason that abusive employers would not permit their care workers to be interviewed in the first place. Another reason might stem from the fact that previous studies of home care workers’ abuse relied on self-administered questionnaires, whereas the present study was based on face-to-face interviews, which were held in the homes of the care recipients. Although the interviews were held in separate rooms, some of the interviewees might have been reluctant to discuss these private issues openly with an interviewer [43]. Another point is that whereas all migrant home care workers who participated in the present study were legally employed, 20% of the migrant home care workers in Israel are undocumented [18]. It is expected that our legally employed participants were at a lower risk for work-related abuse compared with the undocumented migrant care workers [44].

### The vulnerabilities of local home care workers

While we expected to find moderate levels of violations of workers’ rights, the high levels we saw, especially regarding vacation and sick days, are disturbing. This can partly be explained in a few ways. The first is the high relative reports of not getting a written contract, which can lead to not knowing all their working rights. This is especially true for part-time jobs, whereas the rights themselves as well as their scope (e.g., number of vacation days), is not clear and derived from the workload [45].

Another reason might stem from the workers’ socio-economic status (SES). In Israel, as in other developed countries [46], the socio economic status (SES) of home care workers is low, and their employment options are limited [47]. In our sample, 60% of the local live-out care workers were not native Israelis, but veteran immigrants, mainly from the former Soviet Union – a group of low SES in Israeli society [48]. Another source is their command of Hebrew. Although they lived in Israel for many years, many of them did not report a high proficiency in Hebrew. As such, they might not be fully aware of their rights and as a result, might be more vulnerable to violations of these rights by their employers compared with native Israelis [48]. Another source of risk may stem from their financial situation, combined with their age. In Israel, like in other western countries, as people get older they tend to suffer from ageism and have more limited employment options [49], it is possible that some of them would be willing to keep their job despite substantial costs, primarily because losing it can worsen their financial status.

### The vulnerabilities of migrant care workers

In contrast to the low rates of reported work-related abuse, migrant care workers reported high levels of exploitation. Particularly disturbing is that 35% of them reported that they did not have a weekly day off. Although this could reflect a deliberate intention of certain employers to take advantage of their migrant care workers, this could also stem from the close relations between the home care workers and the care recipients. It is well known that over years of work, care workers, become very attached to the care recipient and his or her family [44, 50]. Thus, it is quite possible that some of these workers avoided taking days off because they knew there was no one else to replace them. Financial considerations could also account for these findings as home care workers are often paid extra for not taking a day off. As for living conditions, 12% of the live-in migrant home care workers reported that they did not have their own separate room, and some even reported that they were sharing the care recipient’s bed. We are unable to determine whether this was due to the conditions of the apartment, or at the request of the family, as sometimes happens with cognitively impaired older adults. Whatever the circumstances, sharing a bed is inappropriate, and is liable to lead to abuse [51]. As past research showed that migrant care workers are reluctant to report abuse and exploitation [52], our finding stresses the need for the development of further policy and interventions to protect this already vulnerable population.

Another interesting finding is that certain worker rights violations, such as not receiving all the vacation days, were more prominent than others, such as receiving a written contract and receiving the agreed financial compensation. This could be mainly due to the home care setting, in which working conditions are often blurred [51], especially for part-time workers [53], and for live-in workers [36]. For example, while a live-out care worker may cancel a scheduled home visit due to illness, what could a live-in care worker do when sick? Also, workers who work part-time might not understand their entitlement completely to a pro-rata sick and leave days [42]. Another reason might be that some care workers are deliberately waiving some of their worker’s rights to increase their salary by working longer hours or by not taking all the entitled leave days. The rights waiving can also be the results of the strong relationship which developed between the care recipient and the care worker [54]. When this happens, the care worker may decide to waive certain rights in favor of the care recipient out of caring and affection for the care recipient. These self-imposed violations are no less severe, as the consequences to the wellbeing of the care worker, and as a result the care recipient, can be devastating.

### Comparing the vulnerability of local and migrant care workers

Comparing the incidence of workers’ rights violations and abusive events of live-out local and live-in migrant home care workers, we found that live-in migrant home care workers were 50% more likely to report not receiving paid sick days, 30% more likely to report not receiving vacation days and twice as likely to report emotional abuse compared with local care workers. When added to the rates of violations of workers’ rights that were specific to live-in home care workers, these statistics suggest that migrant home care workers might be at a higher risk for exploitation. Unlike local home care workers, who only work for a couple of hours every day, migrant home care workers live in the home of the most impaired care recipients. This increases the probability of work-related abuse as a function of the time spent with the care recipients [55] and the characteristics of the care recipients - especially those who suffer from cognitive impairments and tend to be more aggressive [56].

As in many other countries, migrant home care workers need to go through a lengthy process to obtain a working permit. While the law allows the home care agencies to collect about 1100 USD as a handling fee from the migrant care workers, the workers often end up paying thousands of dollars as illegal fees to brokers in the host and sending countries [25]. This phenomenon of debt-bondage is widespread worldwide, and many migrant care workers report the need to pay extra money to agents just to obtain a work permit [57]. Because many of them took enormous loans to pay these illegal fees, during their first years of employment most of their salaries are used towards settling these enormous debts [25]. Under these circumstances, leaving an abusive employer is extremely difficult [10].

The high rate of violations of workers’ rights could also arise from the employers’ ignorance regarding migrant workers’ rights. Apparently, certain care recipients and their family members either believe that migrant home care workers are not entitled to all the workers’ rights given to local care workers or they are unaware of these rights and entitlements more generally [6]. This could explain the gap between the relatively small number of reported work-related abuse cases and the high number of reported workers’ rights violations, as the latter might have indicated ignorance or dependency due to the unbalanced power relations [58]. It is important to note that the high rates of workers’ rights violations were found, even though all migrant care workers were legally employed. It is expected that the working conditions of those who are illegally employed are much worse.

While we found a few differences between local and migrant care workers regarding exposure to work-related abuse and exploitation, most of the working conditions and work-related maltreatment and exploitation levels were the same. This is a bit surprising, given the fact that these two populations are different in many ways, including age, origin, level of knowledge of the local language and level of financial distress and workload. On the other hand, they share other attributes, such as the working atmosphere and low socio-economic status.

A few other demographic differences may also contribute to the results. For example, compared to local care workers, migrant care workers reported a lower level of Hebrew knowledge. This creates a fertile ground for violations by the employers [59], as it prevents them from being fully aware of their rights [42]. Indeed, recent reports by the UN shows that many migrant care workers are laboring under contracts they don’t understand, in languages they cannot thoroughly read, or that provide inadequate protection of their rights [60]. This problem persists despite the availability of templates such as the Standards Terms of Employment (STOE). Also, as studies have shown, the language plays a significant role in help-seeking [61, 62]. Language limitations may prevent the migrant care workers from reporting exploitation to officials [36]. Indeed, studies that compared the help-seeking behaviors of locals and immigrants found that the latter typically avoided reporting victimization [63, 64].

Another reason might be the cultural difference between the local and migrant home care workers both regarding willingness to report the abuse and their understanding of the questions. While this questionnaire was translated into English and back-translated and was used in the past with migrant care worker, still a couple of issues arise. For example, we know that in more cooperative societies, such as the Philippines, the willingness to report victimization is more limited [52]. We also know that even after translation and back translation of sensitive topics like abuse, there is still the potential to misinterpret the items [65]. While English is an official language in the Philippines, it is possible that a Tagalog-language questionnaire would have yielded different results.

### Implications for health policy and practice: How can the carers be cared for?

Social workers can play an important part in securing the welfare of care workers [26]. Many services related to home care are coordinated, provided and supervised by social workers [27]. Monitoring the psychosocial needs of older adults [66] and helping to adjust to chronic conditions [27], are among the roles of social workers in the home care setting. Social workers are responsible for the placement of the home care workers within the home and for the welfare of the care recipient as well as the care worker. Ayalon, Kaniel [47] argue that in order for this arrangement to have the best outcomes, both the care recipient and the home care worker should be prepared for it in advance, and they should both be supervised and offered emotional support. It is possible that exploitation could be reduced if both the care recipient and the care worker are prepared properly. In cases of non-deliberate violations, defining the expectations and the roles of the care workers and clarifying the workers’ rights might prove effective. The fact that in some countries care recipients and care workers have inaccurate perceptions regarding the roles of care workers [6, 67, 68], highlights the importance of the social worker in clarifying the roles within this caregiving arrangements. Ongoing follow–up by a social worker is desired as additional demands are likely to occur over time.

The success of social workers in reducing or preventing work-related abuse and exploitation also depends on the active involvement of the government of the host country. It seems that some governments that rely on the work of migrant workers, are reluctant to ensure their basic rights [21] . Israel is not different, as it refuses to sign the international treaty on the welfare of home care workers, which secures rights such as overtime payment and the right to move from one employer to another freely and without any restrictions [46]. Instead, this responsibility is almost completely delegated to the home care agencies that recruit the workers [9]. In addition although the Israeli law determines that a social worker from the home care agency must visit the care recipient “regularly”, in practice such visits are performed only once in 4 months. The visit is focused mainly on the well-being of the care recipient and the quality of the care he or she is given by the migrant home care worker [19]. Because worldwide, most of these agencies are for-profit organizations [68], there is a constant conflict of interests between the welfare of the client and that of the agency [69]. This inherent tension might enhance the obligation dilemma of social workers who work in these agencies as they sometimes may find themselves choosing between their loyalty to the client and their loyalty to the employer [63]. Thus, if more migrant home care agencies were non-profit, the conflict of interests between the welfare of the worker and the agency might have been reduced. However, sufficient involvement of the state in regulation by increasing the frequency the visits made by social workers and external workers’-rights inspectors, might reduce exploitation even in the current for-profit care scheme.

The present study has a number of limitations. Its cross-sectional design prevents reaching cause-and-effect conclusions. Also, only care workers from central Israel were recruited. Home care workers employed in the periphery or in areas where the SES is generally lower might have different experiences. Another limitation has to do with the recruitment method, in which the care recipients or their family members assisted in accessing the care workers, potentially leading to under-representation more severely abused workers. However, receiving lists of migrant care workers from nursing companies was not possible. Another limitation is the reliance on the close-ended questionnaire, which did not allow us to describe examples of abuse qualitatively. Also, future studies in the area of abuse of migrant care workers from the Philippines should include Tagalog-language questionnaires, which will also be culturally adapted [51].

## Conclusions

Our findings demonstrate that whereas both local and migrant home care workers are subject to work-related exploitation, migrant home care workers are particularly vulnerable. Given the prevalence of this caregiving arrangement and the important role of social workers in this setting, the active involvement of the state in regulation by increasing the frequency the visits made by social might reduce exploitation. In addition, implementation of the bilateral agreements like in the construction and agriculture industries, can reduce and even eliminate illegal recruitment fees paid by the migrant care workers.
